# Graphical Trajectory Comparison to Identify Errors in Data of COVID-19: A Cross-Country Analysis

**DOI:** 10.3390/jpm11100955

**Published:** 2021-09-25

**Authors:** Lan Yao, Wei Dong, Jim Y. Wan, Scott C. Howard, Minghui Li, Joyce Carolyn Graff

**Affiliations:** 1 Health Outcomes and Policy Research, College of Graduate Health Sciences, University of Tennessee Health Science Center, Memphis, TN 38163, USA; lyao5@uthsc.edu; 2 College of Medicine, University of Tennessee Health Science Center, Memphis, TN 38163, USA; wdong6@uthsc.edu (W.D.); jwan@uthsc.edu (J.Y.W.); 3 College of Nursing, University of Tennessee Health Science Center, Memphis, TN 38163, USA; showard5@uthsc.edu; 4 College of Pharmacy, University of Tennessee Health Science Center, Memphis, TN 38163, USA; mli54@uthsc.edu

**Keywords:** COVID-19, death rate, disease pattern, error, onset, time lag

## Abstract

Data from the early stage of a novel infectious disease outbreak provide vital information in risk assessment, prediction, and precise disease management. Since the first reported case of COVID-19, the pattern of the novel coronavirus transmission in Wuhan has become the interest of researchers in epidemiology and public health. To thoroughly map the mechanism of viral spreading, we used the patterns of data at the early onset of COVID-19 from seven countries to estimate the time lag between peak days of cases and deaths. This study compared these data with those of Wuhan and estimated the natural history of disease across the infected population and the time lag. The findings suggest that comparative analyses of data from different regions and countries reveal the differences between peaks of cases and deaths caused by COVID-19 and the incomplete and underestimated cases in Wuhan. Different countries may show different patterns of cases peak days, deaths peak days, and peak periods. Error in the early COVID-19 statistics in Brazil was identified. This study provides sound evidence for policymakers to understand the local circumstances in diagnosing the health of a population and propose precise and timely public health interventions to control and prevent infectious diseases.

## 1. Introduction

Despite advances in microbiology and molecular diagnostics, the timely identification of a new infectious disease such as COVID-19, its transmission pattern, the hazard to humans, and case fatality rate, remains challenging [1,2,3,4]. Investigators and clinicians in Wuhan quickly identified the novel coronavirus SARS-CoV-2 and disseminated their findings to the global community but determining the exact date of onset in Wuhan and the dates of initial arrival to other cities has been problematic [1]. Moreover, before mass testing programs were initiated, researchers could determine the exact counts of COVID-19 cases in Wuhan during the first quarter of the pandemic [5]. However, accurate estimation of the date of virus onset in each locale and anticipation of future cases and deaths are necessary to prevent, predict, and mitigate the impact of new potentially lethal communicable diseases.

Since COVID-19 attacked the world, the term “turning point” appears frequently in the news, reports, and publications. From the perspective of epidemiology, the “turning point” means when an infectious disease reaches a point beyond any local ability to control it from spreading more widely [1]. In general, the cases and deaths from the COVID-19 pandemic are expected to decrease after their turning points in a country or region. Unfortunately, new waves of COVID-19 have been observed in many countries and regions. To date, the meaning of the turning point for the development of the pandemic has received scant attention in the research literature. In this study, the turning point indicates the peak of cases or deaths in a single wave of COVID-19.

The turning points of cases and deaths caused by COVID-19 across regions and countries provide a useful tool to reexamine the data in the early stage of the Wuhan outbreak [4,5]. By reviewing and comparing the turning point dates of the COVID-19 pandemic in sampled countries, the disease pattern and trajectory are evident. Despite the multiple waves of disease, some regions and countries, such as China, Hong Kong, South Korea, Switzerland, and Israel, effectively mitigated the epidemic of COVID-19 at the early stages of transmission [2]. From the retrospective investigations of cases, deaths, and calendar days, we found similar patterns in these countries.

We analyzed patterns of turning points of several regions and countries for which robust data on the incidence and timing of COVID-19 cases and deaths were available. We then developed a predictive model to estimate the dates of onset and turning point and applied the model to the early Wuhan pandemic to estimate the date of onset in this and other areas for which early data were less robust due to their timing early in the pandemic.

## 2. Materials and Methods

### 2.1. Data Collection in the Early Stage of the Pandemic

We collected data on COVID-19 cases in China and other countries from official and publicly accessible websites [3,5]. These seven countries (including China) had the largest number of cases at the early stage of the COVID-19 pandemic. We selected seven countries for our study because they were representing European, Asian, North American, and South American continents at the early stage of the pandemic. Analysis of the patterns in these countries would provide useful information on COVID-19 evolution across countries and identify data quality issues to help the public and policymakers to prevent and predict similar disease outbreaks and pandemics in the future. We collected the daily new cases and deaths from the first day of the official reports within regions and countries. The cases and deaths addressed in this study referred to new cases and new deaths. Because COVID-19 was largely under control in China and a few other countries, early data were collected on or before the end of June 2020. When a country did not report the data for new cases or deaths in a single day, the cumulative cases and deaths reported before and after the missing data day were used to estimate the cases and deaths for that specific day. The pandemic period is the interval from the day of the first case to the day in which there were less than 10 cases.

### 2.2. Peak Day of Cases and Deaths

For the peak day of a country, we used the weighted number method [3]. Thus, the peak day of cases is defined as the day with the largest number of average cases of every three, five, and seven days. Similarly, the peak day of deaths is also calculated by the largest number of average deaths of every three, five, and seven days. There will be a maximum of three days of peaks if the peak days from three, five, or seven days are different from each other. For example, if the average deaths of every seven days are the largest number among average numbers of every three, five, and seven days, then the average number of deaths of every seven days will be the deaths peak day. The number of days between the peak days of cases and deaths is defined as the time lag. The peak period is defined as the first day with cases or deaths equal to or more than 40% of the numbers in the cases or deaths peak day to the last day with numbers equal to or more than 40% of the case or death numbers in the cases or deaths peak day, which is 13 days based on previous research [3].

### 2.3. Data Analyses before and after the Peak Day

The number of days of the pandemic before and after the cases peak day and deaths peak day was calculated and compared to rates of SARS-CoV-2 infection and mortality. For cases, the days before and after the cases peak day were calculated respectively and compared to the total days of the study period in different regions/countries. Similarly, the days before and after the deaths peak day were compared to the total number of total days of the study period. The cases peak day and the deaths peak day were not counted in the number of days either before or after the peak days [3]. The term, disease peak, has also been used in the study of characteristics of COVID-19 by other researchers [4,6]. Here we use the term disease peak to define the days of the highest number of cases and deaths as cases peak day and deaths peak day, respectively.

### 2.4. The Time Lag between Peaks of Cases and Deaths

The time lag is defined as the number of days between the cases peak day and the deaths peak day, as calculated based on the paired comparison between three, five, and seven days of the peak day of cases and deaths. The time lags of different countries were calculated. The features of the time lag among countries included the number of days, the proportion of time lag over the total days of the pandemic period, and the proportion of deaths during the time lag over the total deaths. Mathematical calculations in time lag included the numbers in the cases peak day and deaths peak day. The features of the peak period included the duration of time lag, the ratio of time lag to the pandemic period, and the ratio of the cases and deaths to the total cases and total deaths. These features were analyzed among the countries with different pandemic duration and infectious disease outbreak scales.

## 3. Results

### 3.1. Basic Information 

Table 1 summarizes the numeric indicators of cases and deaths of COVID-19 in sampled regions and countries. There was a considerable difference in time lag and numbers of cases and deaths among different countries/regions. The time lag ranged from 0 to 23 days. The case number at peak day ranged from 244 (Huanggang) to 42,941 (Brazil) and the number of deaths at peak day varied from 6 (Huanggang) to 2332 (United States). There was less difference among the days in the peak period of cases and deaths, extending from 7 to 53 and from 22 to 53, respectively.

### 3.2. Case and Death Patterns in Hubei Province

**Figure 1:**
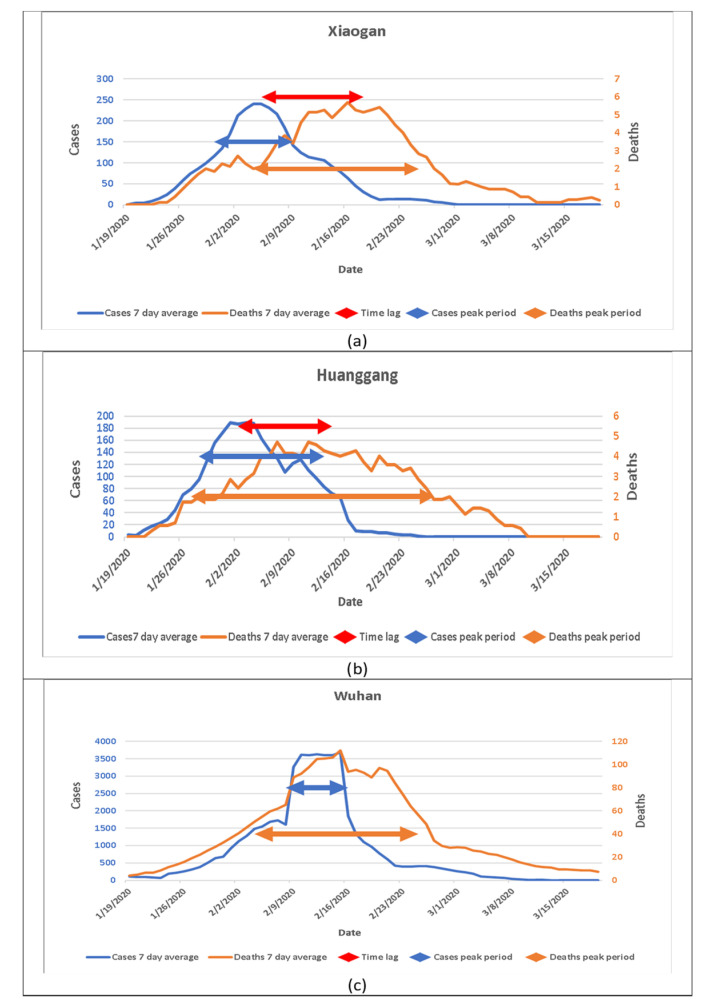
Patterns of cases and deaths from COVID-19 in three cities in China’s Hubei Province from January 19 to March 19, 2020. (**a**) New cases and deaths in Xiaogan; (**b**) New cases and deaths in Huanggang; (**c**) New cases and deaths in Wuhan.

We compared the cases and deaths in Xiaogan, Huanggang, and Wuhan in China’s Hubei province. As shown in Figure 1, we identified peak periods in cases and deaths in each graph and highlighted the time lags in the overlap of cases and deaths.

In Xiaogan, the peak period of cases was between 30 January to 9 February 2020. The turning point or peak day was 5 February 2020. The peak period of deaths was from 4 February to 25 February; the peak day or turning point of deaths was 18 February. The length of the time lag was 13 days (Figure 1a).

In Huanggang, the cases peak period was from 28 January to 13 February; the peak day or turning point of cases was February 2. The deaths peak period was from 28 January to 27 February; the peak day or turning point of deaths was 14 February. The length of the time lag was 12 days (Figure 1b).

The length of time lag represents the average number of days from the onset of symptoms to the deaths in the infected population. Thus, most deaths will not occur on the same day when most cases occur. However, when we examined the data reported from Wuhan, the result was not consistent with other cities. Figure 1c shows the cases peak period (9 February to 15 February) and deaths peak period (4 February to 25 February), but the turning point of cases and deaths were the same (13 February). Therefore, there was no time lag in Wuhan.

### 3.3. Case and Death Patterns in Three Countries with Less Disease Severity

**Figure 2:**
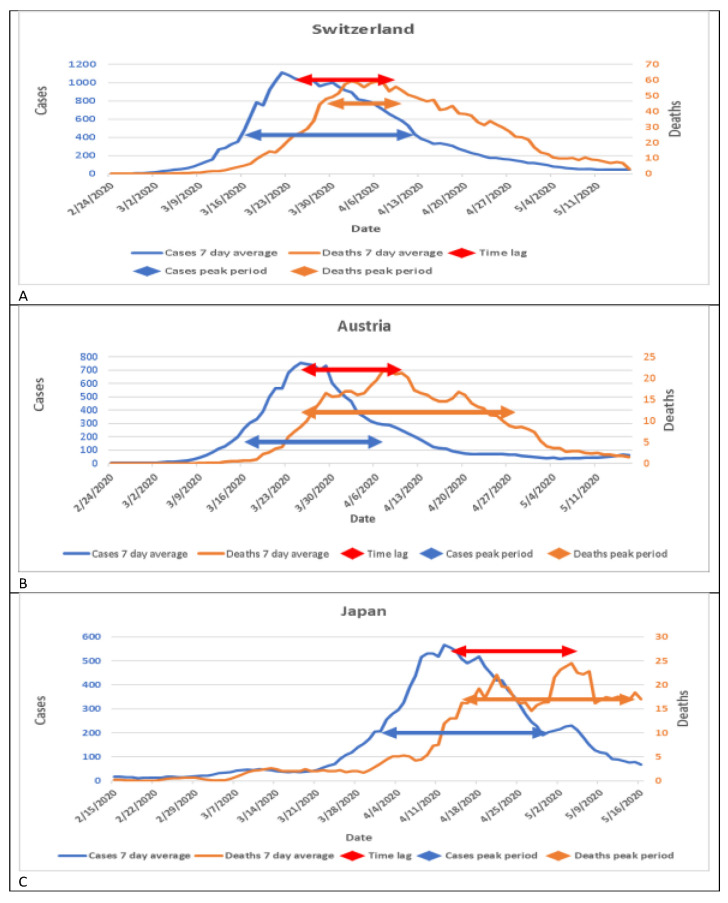
Patterns of cases and deaths from COVID-19 in three countries. (**a**) New cases and deaths in Switzerland; (**b**) New cases and deaths in Austria; (**c**) New cases and deaths in Japan.

To further support the findings that the peak day of cases is before the peak day of deaths, we examined the cases and deaths in Switzerland, Austria, and Japan (Figure 2).

Similar to data from China, cases and deaths in these three countries followed the patterns in the two cities in China. The first peak was the peak of cases, while the last peak was the peak of deaths. The length of time lag was 15 days in Switzerland, 16 days in Austria, and 22 days in Japan.

### 3.4. Case and Death Patterns in Three Countries with Large Pandemics

**Figure 3:**
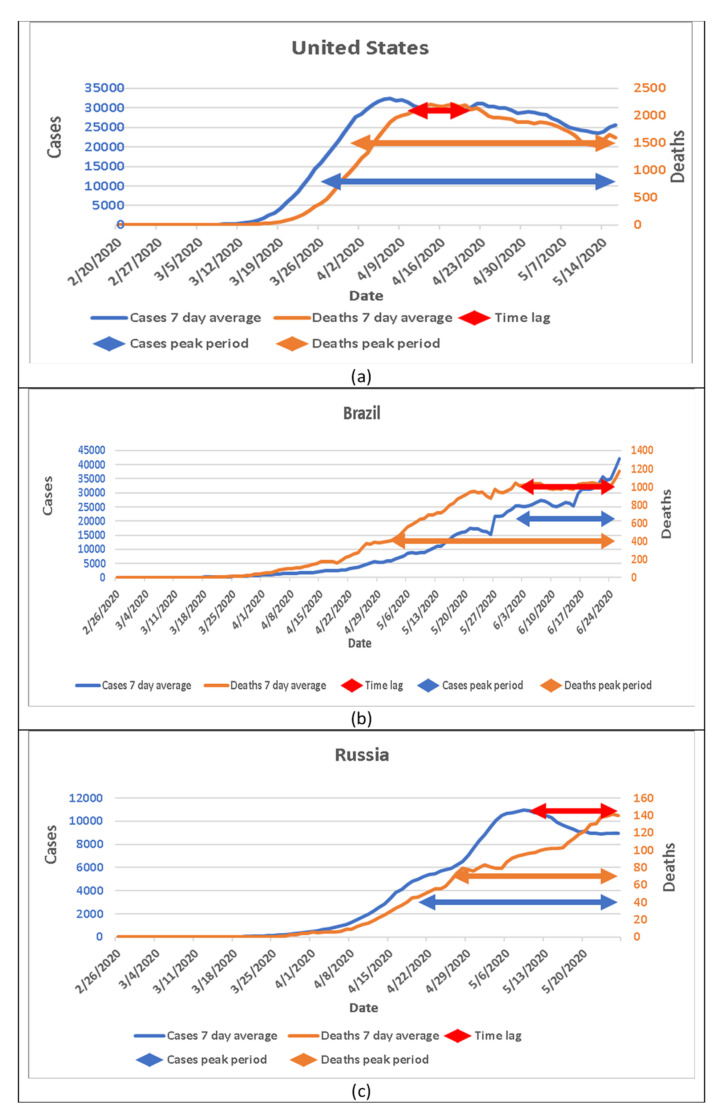
Patterns of death in three different countries with persistently high numbers of COVID-19 cases. (**a**) New cases and new deaths in the United States; (**b**) New cases and new deaths in Brazil; (**c**) New cases and new deaths in Russia.

We chose to examine early data from the United States, Brazil, and Russia, where people are still suffering from the pandemic. COVID-19 in these countries was progressing, but their patterns were different as shown in Figure 3. In the United States, the peak days of cases and deaths could be distinguished, and the time lag was 11 days. In Russia, the situation was approximately the same as the trajectory of Xiaogan and Huanggang in China; the time lag was 16 days. However, in Brazil, the peak of deaths came earlier than the peak of cases; the length of time lag was 23 days (Figure 3).

### 3.5. Patterns of Death Rate around the Peak of Disease Onset

**Figure 4:**
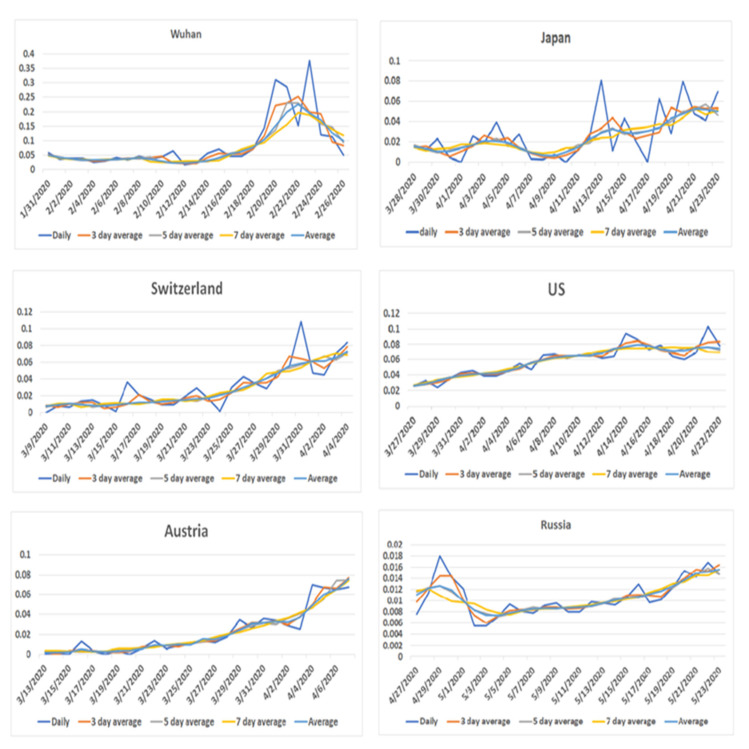
The graphic trajectory comparing COVID-19 death rates between Wuhan and five countries. X-axis represents date of observations. Y-axis indicates the death rate (%). Daily, 3-day average, 5-day average, 7-day average are the average death rates of every day, 3 days, 5 days, and 7 days. The average means the average death rates of daily, 3 days, 5 days, and 7 days in total.

To examine in detail the pandemic onset and the deaths of COVID-19 in Wuhan and to make comparisons across countries, we calculated the case fatality rate according to the 7-day period in Wuhan and sampled countries around the case peak. The case fatality rate of 13 days before and after the onset peak was calculated according to reported information [3]. As shown in Figure 4, although the patterns of case fatality rate in the period around the onset peak in six countries appeared as relatively smooth slopes, the pattern in Wuhan had a drastic increase around 10 days after the disease onset peak. This unique pattern also differed from other regions and countries.

### 3.6. Estimated Peak Day and Cases in Wuhan

According to the data from these countries, several circumstances could explain Wuhan’s divergent results compared to other regions and countries. First, the time lag between peak days of cases and deaths was between 11 to 23 days with an average of 15.5 days. We speculate that the actual peak day of case fatality rate in Wuhan was around January 30, earlier than the reported February 13. The day of early disease onset may also be 30 days before its first reported case [7]. Given that the ratio between reported deaths and cases ranged from 1% to 2% [6], we speculate that the actual number of cases in Wuhan on the peak day was between 6000 to 12,000, while the reported average number of cases over a 7-day was 4800 (Table 1).

### 3.7. Potential Data Collection Errors in Brazil

The surprising finding is that the peak of deaths occurred earlier than the peak of cases in Brazil. The death peak day was 2 June 2020, while the case peak day occurred on 26 June 2020 (Figure 3b). If we assume that the reported deaths are more accurate than those of the cases and the disease pattern of the pandemic in Brazil is the same as other countries, there should be a peak day of cases observed around 20 May 2020. However, reporting bias in the counts of deaths, or the counts of both deaths and cases and improvement in clinical treatment may have led to these findings.

## 4. Discussion

Different countries may show different patterns of cases peak days, deaths peak days, and peak periods. The graphical trajectory analysis reveals significant underestimates in the early reporting of COVID-19 in Wuhan. Such an underestimation may be caused by incomplete data collection in the early stage of the disease, which generally is the case in the early stage of a novel disease [2]. The data collection of COVID-19 outcomes in Wuhan are based on surveillance data with correction for reporting bias. Aggregating data from regional and national sources, we conclude that the official statistics in Wuhan are less than the actual transmission scale of the epidemic, and this finding can be generalized to some countries where the heavy burden of disease has lasted for several months. Implementing mandatory health policies that emphasize social distancing, pooled testing, and wearing masks offers a promise to fight against the pandemic until vaccinations and herd immunity become effective [1].

The statistical confusion around the cases and deaths peak days in Brazil remains a puzzle. In addition to a validation problem in data collection, there are multiple reasons for such a trajectory. First, an error in data collection and analysis, such as statistical analysis or technical errors, could lead to such a result. Second, if the clinical treatment was dramatically improved later, the deaths peak day could occur earlier. Third, if environmental conditions, such as temperature, lead to an early high death rate, and later the death rate decreases because of the high temperature, the deaths peak day may occur earlier than the cases peak day [8]. Furthermore, because the pandemic in Brazil is still not under control, the overall pattern may change in the future.

The differences between surveillance statistics and our estimates may be due to various measurement and health policy limitations. The capacity of testing sites was a major obstacle for detecting cases in Wuhan at the early stage of the epidemic. Citizens with mild symptoms did not have access to testing and healthcare services. Most COVID-19 testing was performed on hospitalized patients, and a few qualified laboratories serving a large population postponed reporting the results. Biases on the number of cases are most likely caused by the large number of asymptomatic and mild cases that did not report themselves or were not tested or diagnosed. Moreover, the overwhelmed healthcare system of Wuhan in the early stages of the outbreak could not provide sufficient services to the COVID-19 patients with mild symptoms. Last but not least, the false positive and false negative results of COVID-19 testing may have contributed to inaccurate data [9,10].

The biases of underestimating the cases and deaths were not limited to Wuhan. Due to the incubation period, asymptomatic carriers, sensitivity and specificity of tests, and testing capacity, underestimating outcomes is common; however, their impact will vary across countries. Even given sufficient testing capacity, both the asymptomatic patients and false-negative results would underestimate the number of cases [11,12]. Essentially, policymakers and healthcare professionals may encounter a substantial underestimate of the actual number of cases and thus misunderstand the development of the pandemic. We hope that this straightforward graphical trajectory approach can be used for similar conditions to eliminate bias.

In addition, these biases involve time-dependent reporting of cases and deaths and time lags in COVID-19 outcomes. In the situation of estimating COVID-19 time lag, we identify the biases and their possible relevance. Further, we provide a partially corrected estimation of these biased data from time lag and incomplete reporting of cases and deaths. This study shows that contact tracing of infected individuals despite the presence of symptoms will alleviate bias by controlling the correlation between diagnosis and deaths.

Researchers have established models to predict the progression of COVID-19 [11], such as estimation of cases and deaths based on algorithms [3], deep learning models for predicting severe progression [13], and prospective validation studies of prognostic biomarkers to predict adverse outcomes [14]. Our study is an evidence-based comparison and prediction, which is different from typical statistical models.

In this article, we discuss the remaining situations that need to be considered. First, all data were derived from website data reported by each region or country. Variations in the measurement of cases and deaths in the early stage of the pandemic may exist, which is one reason there is no time lag in Wuhan. Second, countries with progressive COVID-19 require long-term attention as the existing data lead to a limited understanding of the situation. Third, studies have demonstrated that the durations from being infected with COVID-19 to death vary from 1 to 21 days [7,15] while the death peak appears about 13 days after infection [7]. The causes of the same peaks of cases and deaths in Wuhan could be multifold, including the urgent shortage of medical facilities, which led to the immediate or excessive deaths, the reporting biases of cases and deaths, and the insufficient contact tracing of infected individuals. One of the errors in early Wuhan data was its high mortality rate which had been calculated as high as 20% at the early stage of the epidemic [3]. If the death peak was earlier than that of the case peak, this is evidence that the data were not valid.

As shown in our findings, graphical trajectory comparison is capable of identifying potential errors in data collection and reporting at the early stage of infectious diseases. If this approach is used to monitor the reported data from different regions of a country or among different countries, errors may be identified and corrected to provide more reliable information for the public and governments to implement timely public health interventions to control and prevent infectious diseases.

## 5. Conclusions

Our simulated graphical trajectory method identifies statistical biases in surveillance data. This approach incorporates all sources of available data and provides a robust method to characterize the time course of an infectious disease. Regions and countries beginning with high mortality rates from the COVID-19 epidemic will suffer a long, painful period of the disease epidemic. Where the mortality rate is relatively high, healthcare professionals should prepare for a longer period of fighting this pandemic. Data quality is key to case fatality rate estimation which is needed by policymakers to make correct and timely critical decisions.

## Figures and Tables

**Table 1:** Summary of Indicators of Cases and Deaths of Sampled Regions and Countries using data ^1^ of an average of 7-day.

Indicators	Xiaogan	Huanggang	Wuhan	Switzerland	Japan	Austria	United States	Brazil	Russia
Time lag	13	12	0	15	22	16	7	−23	16
Cases in peak day	424	244	3910	1100	615	796	32,901	42,941	11,028
Deaths in peak day	7	6	88	57	24	25	2332	1165	140
Cases in peak period	1846	2578	25,393	22,890	11,065	10,449	1461,040	903,395	330,374
Deaths in peak period	64	110	1880	1594	617	491	86,251	48,766	3252
Cases in time lag	1846	1815	0	14,794	8330	7659	241,640	723,396	154,751
Deaths in time lag	64	54	0	643	428	245	17,263	24,831	1806
Cases after peak day	1533	1638	32,994	23,098	10,232	10,613	1,039,256	0	152,654
Total cases	3419	2884	50,860	30,572	16,237	16,201	1,516,575	1,280,063	362,380
Deaths after peak day	58	56	1036	1114	238	356	55,330	23,562	174
Total deaths	128	125	2606	1879	725	629	90,324	56,109	3807
Cases peak period	11	17	7	27	25	18	53	32	39
Deaths peak period	22	31	22	36	35	29	47	53	33
Peak cases/total cases	0.124	0.894	0.499	0.749	0.681	0.645	0.963	0.706	0.912
Peak deaths/total deaths	0.055	0.880	0.721	0.848	0.851	0.781	0.955	0.869	0.854
Cases after peak/total cases	0.448	0.568	0.649	0.756	0.630	0.655	0.685	0.000	0.421
Deaths after peak/total deaths	0.453	0.448	0.398	0.593	0.328	0.566	0.613	0.420	0.046
Cases in time lag/total cases	0.540	0.629	0.000	0.484	0.513	0.473	0.159	0.565	0.427
Deaths in time lag/total deaths	0.500	0.432	0.000	0.342	0.590	0.390	0.191	0.443	0.474

^1.^ Note: The data of Wuhan, Huanggang, and Xiaogan were collected from the Health Commission of Hubei Province at http://wjw.hubei.gov.cn/fbjd/dtyw/ (accessed on 30 June 2020). The data of Switzerland, Japan, Austria, the United States, Brazil, and Russia are available in the repository from Worldometers at https://www.worldometers.info/coronavirus/ (accessed on 30 June 2020).

## Data Availability

The data of Wuhan, Huanggang, and Xiaogan were collected from the Health Commission of Hubei Province at http://wjw.hubei.gov.cn/fbjd/dtyw/ (accessed on 30 June 2020). The data of Switzerland, Japan, Austria, the United States, Brazil, and Russia are available in the repository from Worldometer at https://www.worldometers.info/coronavirus/ (accessed on 30 June 2020).
